# Non-curative therapies and their impact on the prognosis of patients with myelodysplastic syndromes– a retrospective matched-pairs analysis

**DOI:** 10.1007/s00277-025-06485-w

**Published:** 2025-06-27

**Authors:** Katharina Anna Rauhe, Annika Kasprzak, Felicitas Schulz, Kathrin Nachtkamp, Corinna Strupp, Andrea Kündgen, Sascha Dietrich, Karin Mayer, Katharina S. Götze, Wolf-Karsten Hofmann, Aristoteles Giagounidis, Norbert Gattermann, Ulrich Germing

**Affiliations:** 1 Department of Orthopedics, Hospital Bielefeld-Mitte, Medical School and University Medical Center Ostwestfalen-Lippe, Bielefeld University, Bielefeld, Germany; 2 Department of Hematology, Oncology and Clinical Immunology, University Hospital Duesseldorf, Duesseldorf, Germany; 3 Department III of Internal Medicine, University Hospital Bonn, Bonn, Germany; 4 Department of Medicine III, Technical University of Munich School of Medicine and Health, Munich, Germany; 5 Department of Hematology and Oncology, Medical Faculty Mannheim, Heidelberg University, Mannheim, Germany; 6 Department of Oncology, Hematology and Palliative Care, Marien-Hospital, Duesseldorf, Germany

**Keywords:** IPSS-R, Prognosis, Myelodysplastic syndromes, Non-curative therapies

## Abstract

**Abstract:**

Allogeneic stem cell transplantation (SCT) remains the only curative therapy for patients with high-risk myelodysplastic syndromes (MDS). Due to age, comorbidities, or lack of urgency, many receive only palliative therapies to improve quality of life. Some patients remain untreated due to a lack of symptoms or low progression risk. Data on the impact of palliative therapies on overall survival (OS) and leukemia-free survival (LFS) are limited. Using the Düsseldorf MDS Registry, we compared outcomes of patients receiving red blood cell transfusions (RBCT) alone to the outcome of patients receiving RBCT combined with iron chelation therapy (ICT), erythropoietin (EPO), antithymoglobulin (ATG), or lenalidomide (LENA). Matched-pairs analysis was conducted using age, gender, and prognostic scores (Revised International Prognostic Scoring System or Chronic Myelomonocytic Leukemia-specific Prognostic Scoring System). ICT-treated patients (*n* = 85) had significantly improved OS (70 vs. 21 months, *p* < 0.001) and lower 5-year AML progression (3.5% vs. 28.2%, *p* < 0.001). Similar benefits were seen with EPO (*n* = 210; OS: 63 vs. 24 months, *p* < 0.001; AML: 5.7% vs. 19%, *p* = 0.007) and LENA (*n* = 30; OS: 92 vs. 57 months, *p* = 0.049; AML: 0% vs. 16.7%, *p* = 0.024). ATG (*n* = 11) showed no significant improvement in OS (79 vs. 64 months) or AML progression (0% vs. 18.2%). While recognizing the limitations of matched-pairs analysis versus randomized trials, our findings indicate a survival benefit from ICT, EPO, or LENA versus RBCT alone. The year of diagnosis did not independently affect OS or LFS. These results support the use of selected palliative therapies to improve long-term outcomes in MDS patients.

**Key points:**

- Treating patients with myelodysplastic syndromes with non-curative therapies beyond red blood cell transfusions like iron chelation therapy, erythropoietin, or lenalidomide has a positive impact on overall survival and leukemia-free survival.

## Introduction

Myelodysplastic syndromes (MDS) are a heterogenous group of diseases correlated with dysplasia in clonal hematopoietic stem cells of the bone marrow (BM). They are often accompanied by peripheral blood cytopenia like anemia. MDS typically manifest in older adults, with a median age at diagnosis of approximately 70 years [1], and bear the risk of disease progression to acute myeloid leukemia (AML). A distinct hematologic entity closely related to MDS is chronic myelomonocytic leukemia (CMML), characterized by overlapping features of both myelodysplastic and myeloproliferative neoplasms. Over time and under exposure to risk factors like benzene, cytotoxic agents, or ionizing radiation, genetic alterations may occur in hematopoietic stem cells, leading to disturbed hematopoiesis. These alterations can be detected through morphological, cytogenetic, and molecular analyses of the BM. Prognostic scoring systems like the Revised International Prognostic Scoring System (IPSS-R) or CMML-specific Prognostic Scoring System (CPSS) integrate these findings along with peripheral blood parameters to estimate overall survival (OS) and leukemia-free survival (LFS) [2, 3]. The MDS-specific Comorbidity Index (MDS-CI) is an instrument for estimating the risk of MDS-independent death [4]. Currently, allogeneic stem cell transplantation (SCT) remains the only potentially curative treatment for MDS and CMML but is not suitable for most patients due to age, comorbidities, or other contraindications. For most patients, therapy strategies are part of a palliative situation and intend to be supportive. In high-risk MDS patients the primary objective is curation via SCT aiming at extending survival, even at the cost of reduced quality of life during treatment. In contrast, for low-risk cases, the focus shifts toward improving quality of life, without an expectation of prolonging OS or LFS. In fact, asymptomatic patients with low-risk MDS may remain untreated without requiring MDS-specific therapy at all [5]. Common therapeutic options include red blood cell transfusions (RBCT), iron chelation therapy (ICT), erythropoietin (EPO), antithymoglobulin (ATG), lenalidomide (LENA), hypomethylating agents (HMA), and luspatercept. In this retrospective study we aimed to assess OS and LFS in patients who were ineligible for SCT, comparing cohorts of patients who received ICT, EPO, ATG, or LENA with patients treated with RBCT alone. Our objective was to investigate whether predominantly symptom-oriented therapies could provide also prognostic benefit in patients with MDS.

## Methods

### Patients

This study was based on data from patients included in the German MDS Registry, coordinated in Düsseldorf. Patients diagnosed with MDS or CMML were eligible for inclusion. Clinical data were retrospectively retrieved and supplemented with information from the internal hospital’s documentation and data provided by cooperating hospitals and private practices. The observational period extended from the first recorded diagnosis in 1982 to 2023. Data collected primarily covered hematological parameters at the time of initial diagnosis, administered treatments, comorbidities, and outcomes such as progression to AML and OS. A total of 3,850 patients were included in the dataset, of whom 55.7% (*n* = 2,144) were male and 44.3% (*n* = 1,706) were female. The median age at diagnosis was 71 years (range: 18–104 years). The median OS was 23 months (range: 0–477 months). Secondary AML developed in 12.5% (*n* = 481) of patients after a median of 14 months (range: 0–229 months). For the 3,097 patients diagnosed with MDS at initial diagnosis, IPSS-R-based prognostic classification was available in 42.8% (*n* = 1,326), with the following distribution:

- Very low risk: 3.8% (*n* = 118).
- Low risk: 19.1% (*n* = 591).
- Intermediate risk: 12.9% (*n* = 399).
- High risk: 5.4% (*n* = 168).
- Very high risk: 1.6% (*n* = 50).

For the 437 patients initially diagnosed with CMML, the CPSS was used. Complete data were available for 43.5% (*n* = 190), and risk classification was as follows:

- Low risk: 8.5% (*n* = 37).
- Intermediate-1 risk: 14.6% (*n* = 64).
- Intermediate-2 risk: 19.9% (*n* = 87).
- High risk: 0.5% (*n* = 2).

The primary focus of this study was on patients who received low-risk therapies, in total 57.7% (*n* = 2,221). Additionally, patients who underwent cytoreductive therapy (cytarabine or hydroxyurea; *n* = 69 [1.8%]), HMA (azacitidine or decitabine; *n* = 169 [4.4%]), intensive chemotherapy (*n* = 227 [5.9%]), or allogeneic SCT (*n* = 206 [5.4%]) were documented. For 24.9% (*n* = 958), no MDS-specific therapy was recorded during the observation period. Within the low-risk therapy group:

- 73.4% (*n* = 1,630) received transfusions only (mean year of diagnosis: 1998, interquartile range (IQR): 1988–2007).
- 3.9% (*n* = 87) received additional ICT (mean year of diagnosis: 2006, IQR: 1998–2009).
- 10.2% (*n* = 226) received EPO (mean year of diagnosis: 2012, IQR: 2007–2016).
- 0.5% (*n* = 12) received ATG (mean year of diagnosis: 2007, IQR: 2004–2008).
- 1.6% (*n* = 35) received LENA (mean year of diagnosis: 2011, IQR: 2005–2014).
- 10.4% (*n* = 231) received luspatercept, granulocyte colony-stimulating factor, or combinations of the above (mean year of diagnosis: 2009, IQR: 2004–2015).

Anemia is the most common cytopenia in MDS. As indicated in brackets, the non-curative approach to the treatment of MDS has changed over the years with the clinical application of therapies aimed at reducing patients’ transfusion dependency.

### Statistical analysis

To identify variables affecting OS and LFS, the following statistical tests were applied: Pearson’s χ² test for associations between categorical variables, median test for independent k-samples, and stepwise forward Cox regression analysis. Due to the retrospective nature of the dataset, a matched-pairs analysis was used. Cases were defined as patients who received RBCT in combination with one of the following: ICT, EPO, ATG, or LENA. These were compared with control patients who received RBCT only. Matching was based on known outcome-relevant variables: identical IPSS-R/CPSS risk group (with no variation; for CMML, hemoglobin levels as additional criteria a ± 2 g/dl difference was allowed), age at diagnosis (± 5 years), and gender (male or female). OS and LFS differences between case and control groups were evaluated using Kaplan–Meier survival analysis. A significance threshold of α = 0.05 and 95% confidence intervals (CI) were applied throughout.

## Results

### Matching criteria

To identify variables that effect OS or the probability of secondary AML transformation, univariate analyses were performed. The categorical variables examined included gender, year of diagnosis, age, WHO 2016 subtype, MDS-CI, IPSS-R, CPSS, karyotype, BM fibrosis, BM cellularity, BM blast percentage, hemoglobin concentration (Hb), absolute neutrophil count, platelet count, lactate dehydrogenase, and treatment category. Except for treatment category, all variables were assessed at the time of diagnosis. Cox proportional hazards models were employed to assess the impact of the variables on OS and AML progression. For patients with MDS, multivariate analysis identified IPSS-R (mean HR 1.434; CI [1.312–1.569] for ordinal variable: very low vs. low vs. intermediate vs. high vs. very high risk), gender (HR 0.710; CI [0.603–0.836] for dichotomous variable: male vs. female), age (mean HR 1.474; CI [1.358–1.599] for ordinal variable: 18–62 vs. 63–70 vs. 71–77 vs. 78–104 years old), and treatment category (mean HR 0.930; CI [0.876–0.987] for categorial variable: BSC vs. low-dose chemotherapy vs. HMA vs. high-dose chemotherapy vs. allogeneic SCT) as independent predictors of OS. Other variables were either components of the IPSS-R or not statistically significant in stepwise analyses. Regarding AML transformation, age and gender were not statistically significant. IPSS-R (mean HR 1.1; CI [1.063–1.137] for ordinal variable as above) and treatment category (mean HR 1.465; CI [1.383–1.553] for categorial variable as above) remained the most predictive factors. For patients with CMML, OS was significantly influenced by CPSS (mean HR 1.592; CI [1.106–2.292] for ordinal variable: low vs. intermediate-1 vs. intermediate-2 vs. high risk), treatment category (mean HR 0.747; CI [0.639–0.872] for categorial variable as above), and hemoglobin (HR 1.734; CI [1.090–2.757] for dichotomous variable: above vs. under 9 g/dl). AML progression in CMML was significantly affected by CPSS (mean HR 1.684; CI [1.051–2.699] for ordinal variable as above) and treatment category (mean HR 1.260; CI [1.021–1.555] for categorial variable as above). Based on these results, the matching variables for a matched-pairs analysis were defined. In total, 336 patients were identified who received blood products and one additional low-risk therapy (ICT, EPO, ATG, or LENA) throughout their disease progression. They are hereafter referred to as cases. These were matched with 336 so-called controls who received blood products only. Notably, 61.5% (*n* = 207) of controls were diagnosed before 2000, compared to only 8.9% (*n* = 30) of the cases. The year of diagnosis seemed to be the main difference between cases and controls and most likely the reason for the different treatment while there was no significant difference in the blood cell count or comorbidity burden measured via the MDS-CI at the time of diagnosis.

### Matched-pairs analyses

#### ICT

Based on age, gender, and IPSS-R (14.3% very low, 46.9% low, 32.7% intermediate, 6.1% high risk) (or alternatively CPSS and Hb), with tolerances of ± 5 years in age and ± 2 g/dl in Hb, 85 matched pairs (97.6%) were formed from 87 eligible cases. Kaplan-Meier analysis revealed an OS of 70 months for cases (CI [57.7–82.3]) vs. 21 months for controls (CI [16.6–25.4]) (Log-rank χ²(1) = 14.726, *p* < 0.001). AML progression occurred in 1.2% (*n* = 1) of cases vs. 27% (*n* = 23) of controls within two years, and 3.5% (*n* = 3) vs. 28.2% (*n* = 24) within five years (Log-rank χ²(1) = 15.229, *p* < 0.001). Stratified analyses by MDS-CI risk group showed significantly better OS in the low-risk group among cases (70 vs. 19 months; Log-rank χ²(1) = 6.219, *p* = 0.013). For intermediate risk, OS was 61 vs. 23 months (Log-rank χ²(1) = 3.788, *p* = 0.052, Breslow χ²(1) = 7.459, *p* = 0.006, Tarone-Ware χ²(1) = 5.753, *p* = 0.016). In high-risk patients, survival curves crossed, and no statistically significant difference was found.

#### EPO

Using the same matching criteria (IPSS-R: 13.8% very low, 50.8% low, 28.5% intermediate, 6.9% high risk), 210 pairs (92.9%) were formed from 226 cases receiving EPO. OS was 63 months for cases (CI [52.5–73.5]) vs. 24 months for controls (CI [17.2–30.8]) (Log-rank χ²(1) = 23.761, *p* < 0.001). AML progression occurred in 2.9% (*n* = 6) of cases vs. 16.2% (*n* = 34) of controls within two years, and 5.7% (*n* = 12) vs. 19% (*n* = 40) within five years (Log-rank χ²(1) = 7.362, *p* = 0.007). Stratified analyses by MDS-CI risk group showed significantly better OS in the low-risk group among cases (95 vs. 24 months; Log-rank: χ²(1) = 6.789, *p* = 0.009; Breslow: χ²(1) = 13.391, *p* < 0.001; Tarone-Ware: χ²(1) = 11.155, *p* < 0.001). For intermediate risk, OS was 52 vs. 17 months (Log-rank: χ²(1) = 5.341, *p* = 0.021; Breslow: χ²(1) = 14.796, *p* < 0.001; Tarone-Ware: χ²(1) = 10.882, *p* < 0.001). For high risk, OS was 50 vs. 26 months (Log-rank: χ²(1) = 4.186, *p* = 0.041; Breslow: χ²(1) = 3.390, *p* = 0.066; Tarone-Ware: χ²(1) = 3.787, *p* = 0.052).

#### ATG

Using the same matching criteria (IPSS-R: 12.5% very low, 75% low, 12.5% intermediate risk), 11 pairs (91.7%) were formed from 12 cases receiving ATG. Kaplan-Meier analysis revealed a median OS of 79 months (CI: 0–166.357) in the case group and 64 months (CI: 18.047–109.953) in the control group. Log-rank, Breslow and Tarone-Ware did not show a significant difference. AML progression occurred in 18.2% (*n* = 3) of controls within the first two years and remained stable over five years. No AML was observed in the ATG-treated group. The log-rank test demonstrated a non-significant trend: χ²(1) = 3.098, *p* = 0.078.

#### LENA

Using the same matching criteria (IPSS-R: 4.2% very low, 62.5% low, 33.3% intermediate risk), 30 matched pairs (85.7%) were formed from 35 cases receiving LENA. Kaplan-Meier analysis showed an OS of 92 months (CI: 37.443–146.557) in the LENA group vs. 57 months (CI: 18.927–95.073) in controls. The log-rank test indicated a statistically significant difference: χ²(1) = 3.862, *p* = 0.049; Tarone-Ware: χ²(1) = 4.189, *p* = 0.041. No AML progression occurred within two years in the LENA group, compared to 10% (*n* = 3) in the control group. After five years, AML occurred in 16.7% (*n* = 5) of controls and none of the cases. The difference in AML-free survival was statistically significant: χ²(1) = 5.062, *p* = 0.024.

### Temporal trends

Over the last forty years, the unadjusted OS of patients diagnosed with MDS has improved (OS before 1990: 19 months (CI: 15.784–22.216) vs. OS in the 1990s: 24 months (CI: 19.908–28.092) vs. OS in the 2000s: 42 months (CI: 37.397–46.603) vs. OS in the 2010s: 50 months (CI: 44.199–55.801) vs. OS in the 2020s: not yet reached). Most patients receiving transfusions only were diagnosed before 2005 (78.6%), whereas the majority receiving additional supportive therapy (ICT, EPO, ATG, or LENA) were diagnosed after 2005 (78.9%). A multivariate Cox regression including therapy type (transfusions only vs. transfusions plus supportive therapy), IPSS-R risk, and diagnosis decade (pre-1990, 1990 s, 2000 s, 2010 s, 2020 s) revealed a significant impact of therapy (*p* < 0.001) and IPSS-R (*p* = 0.007), but not diagnosis timing (*p* = 0.38) on OS. For AML transformation, therapy type (*p* < 0.001) and IPSS-R (*p* < 0.001) were again significant, while diagnosis timing had no significant effect (*p* = 0.979).

## Discussion

Retrospective matched-pairs analyses are not of the same quality as randomized controlled trials and may be subject to bias due to limited retrospective data availability and the selection of matching-criteria that may not represent all aspects of the treatment decision or health status. The use of data from a longer observation period made it possible to include treatments (RBCT only) in the analyses that are no longer preferred today, as the clinical benefit of complementary supportive therapies is already recognized, even if the data on OS and LFS are limited. This study aimed to evaluate the impact of ICT, EPO, ATG, and LENA on OS and the risk of progression to AML in patients with MDS who are ineligible for allogeneic SCT or had an MDS profile where SCT was not initially indicated.

ICT showed a significant benefit in both OS and the risk of progression to AML in the treatment group compared to controls. Subgroup analyses based on the MDS-CI revealed that patients with low to intermediate comorbidity burdens benefited more than those with a high burden. These findings are in line with previous research by Neukirchen et al. [6]. Since anemia is a dominant impairment in MDS, many patients become chronically transfusion-dependent, often resulting in secondary iron overload and end-organ damage, a condition known as iatrogenic hemochromatosis [7]. Metzgeroth et al. [8] demonstrated a significant reduction in hepatic and cardiac iron concentrations with deferasirox in a phase II study using T2*-weighted magnet resonance imaging, providing a plausible mechanism for improved OS due to reduced cardiac risk. Furthermore, murine models suggest iron overload may promote leukemogenesis through oxidative stress and related mutagenic effects [9]. As ICT also reduces medullary iron levels [8], the observed reduction in AML progression risk among MDS patients with iron overload appears biologically reasonable.

EPO therapy was associated with a significant improvement in OS and reduced AML progression risk. Patients with low or intermediate comorbidity burdens appeared to benefit. EPO stimulates hematopoiesis in patients with endogenous EPO deficiency, reducing or eliminating transfusion requirements [10, 11]. This, in turn, minimizes transfusion-related risks and prevents secondary iron overload. Additionally, in the context of patient blood management strategies [12], EPO helps conserve limited blood resources, reserving transfusions for emergency situations. While improved OS with EPO use is well-documented [13], especially in low risk MDS [14], the mechanism by which EPO may reduce AML progression risk remains unclear. A hypothesis like that of ICT is plausible: reduced transfusion burden and avoidance of transfusion-induced iron overload.

In this study cohort, no significant differences in OS or AML progression risk were observed between patients receiving ATG and those managed symptomatically with transfusions. However, this finding is limited by the small sample size (11 in each group), which only allows detection of large effects. Nachtkamp et al. [15] previously reported a positive impact of ATG on OS without addressing AML progression. Given the assumed autoimmune component of some MDS cases [16], parallels have been drawn from the treatment of aplastic anemia, where ATG is approved [17]. Several studies have demonstrated that ATG, particularly when combined with cyclosporine a, can induce hematologic responses and transfusion independence in MDS patients [18–20]. A phase II trial also reported remissions and transfusion independence with ATG [21]. Early studies suggest that the effectiveness of ATG in low-risk MDS may depend on the specific MDS subtype [22, 23].

LENA showed a positive effect on OS and AML-free survival in patients with del(5q) MDS. In a phase III study, Fenaux et al. [24] demonstrated prolonged OS and LFS in patients who achieved transfusion-independency with LENA, as well as prolonged OS in patients with a cytogenetic response to LENA. Schuler et al. [25] were able to demonstrate a significant reduction or even loss of transfusion dependency in patients with del(5q) MDS treated with LENA. Studies by Kündgen et al. [26] demonstrated reduced transfusion dependence and cytogenetic responses in this subgroup, including a diminished presence of del(5q) clones. Mossner et al. [27] found that TP53 loss-of-function mutations in del(5q) MDS patients significantly worsen OS and AML-free survival. Mechanistically, LENA induces ubiquitination and degradation of casein kinase 1A1, encoded within the deleted 5q region [28]. This gene acts as a tumor suppressor and its haploinsufficiency may promote proliferation of malignant clones [28]. LENA-mediated inactivation leads to apoptosis in these mutated cells [28].

In summary, therapies such as ICT, EPO, and LENA are beneficial for MDS patients not eligible for SCT, improving OS and reducing the risk of AML progression. Due to the limited sample size, conclusions regarding ATG remain tentative and warrant validation in larger cohorts. Future subgroup analyses are needed to better identify which patient populations benefit most from specific supportive therapies and whether combination approaches may offer additional advantages. Further research into the cellular mechanisms by which ICT and EPO influence progression risk is also warranted. Emerging data on somatic mutations have greatly enhanced our understanding of disease progression in MDS. The Molecular International Prognostic Scoring System (IPSS-M), which incorporates molecular markers, allows more accurate prognosis than IPSS-R and may guide the timely use of aggressive therapies in high-risk patients before clinical deterioration precludes curative options [29, 30]. Conversely, it also allows better identification of low-risk patients who may be managed conservatively, avoiding overtreatment and its associated complications [31]. Detecting AML-associated mutations through sequencing of BM aspirates can aid early recognition of disease transformation, enabling prompt intervention [32–34].

Overall, the management of MDS has advanced considerably, and supportive care strategies are essential components of therapy that should be actively considered in eligible patients. Ongoing efforts aim to individualize therapy and improve long-term outcomes through a personalized precision medicine approach.

## Data Availability

The data that support the findings of this study are not openly available due to reasons of sensitivity and are available from the corresponding author upon reasonable request.
